# Impact of Prior Metabolic and Bariatric Surgery on Outcomes of Total Knee and Total Hip Arthroplasty: A Systematic Review and Meta-analysis

**DOI:** 10.34172/aim.34291

**Published:** 2025-08-01

**Authors:** Mahta Shari’at Moghani, Ali Esparham, Mohammad Mahdinezhad Kashani, Negar Einafshar, Mahsa Radboy, Mohammad Javad Ghamari, Tooraj Zandbaf

**Affiliations:** ^1^Students Research Committee, MMS.C., Islamic Azad University, Mashhad, Iran; ^2^Faculty of Medicine, Mashhad University of Medical Sciences, Mashhad, Iran; ^3^Department of Orthopedic, MMS.C., Islamic Azad University, Mashhad, Iran; ^4^Innovative Medical Research Center, MMS.C., Islamic Azad University, Mashhad, Iran; ^5^Department of General Surgery, MMS.C, Islamic Azad University, Mashhad, Iran

**Keywords:** Arthroplasty, Complication, Metabolic and bariatric surgery, Obesity, Osteoarthritis, Outcome

## Abstract

**Background:**

The current study evaluated the effects of prior metabolic and bariatric surgery (MBS) on complications after total knee/hip arthroplasty (TKA/THA). We performed a meta-analysis to assess the impact of prior MBS on TKA/THA outcomes.

**Methods:**

Our systematic search was conducted on PubMed, Embase, Scopus, and Web of Science until September 2024. Thirteen studies were included in total, one of which was an RCT, and the others were retrospective studies.

**Results:**

According to our findings, MBS was linked to decreased risk of peri-prosthetic joint infection in 853 MBS vs. 835 non-MBS patients (OR: 0.55, 95% CI: 0.31, 0.97, *P* value: 0.04), deep venous thromboembolism in 1074 MBS vs. 11948 non-MBS patients (OR: 0.50, 95% CI: 0.28, 0.86, *P* value: 0.01), and shorter length of hospital stay in 2,221 MBS vs. 12,201 non-MBS patients (mean difference: -0.42 days, 95% CI: -0.71, -0.13, *P* value<0.001) after TKA/THA. Aseptic loosening, blood transfusion, peri-prosthetic fracture, postoperative manipulation, readmission, reoperation, dislocation, pulmonary thromboembolism, revision, and wound complications were not significantly associated with MBS.

**Conclusion:**

MBS prior to TKA/THA can considerably reduce some post-operative complications, such as the risk of PJI, DVT, and LOS at the hospital. It can be offered to patients with severe obesity before undergoing TKA/THA.

## Introduction

 Over the past 35 years, the prevalence of overweight and obesity has increased significantly to the point where over one-third of the world’s population is currently considered overweight or obese.^1^ Additionally, a significant frequency of various chronic diseases has been linked to overweight and obesity, highlighting the necessity of weight management strategies that treat both excess body weight and related consequences.^2,3^

 Obesity, as an ongoing significant pandemic, links causally to hip and knee osteoarthritis (OA).^3-7^ Also, obesity is the most well-established modifiable risk factor for OA, the most common chronic joint disease, at the individual level.^8,9^ Additionally, OA as a cause of disability progresses more rapidly and severely in patients with obesity, who can greatly benefit from total joint arthroplasty (TJA).^10,11^ Over time, the number of younger and overweight patients undergoing both total hip arthroplasty (THA) and total knee arthroplasty (TKA) is increasing.^12^ Patients with obesity face higher perioperative complications, such as a longer length of hospital stay (LOS), the possibility of earlier revision, and higher costs after TKA/THA due to their association with another comorbid disease. Similarly, it was reported that they have higher infection and dislocation rates and lower implant survivability and functional scores after surgery compared to non-obese individuals.^3-6,10,13-16^ Thus, for orthopedic surgeons, obesity is a common complicating factor that presents several difficulties.^10,17^

 It has been noted that MBS can be considered a viable option for patients with obesity and obesity-related comorbidities before THA and TKA.^18,19^ The impact of weight loss through MBS prior to TJA on postoperative complications remains a subject of debate within the academic community. To the best of our knowledge, few systematic studies have directly compared the prognosis of TJA in obese patients with and without prior MBS. Existing studies with similar objectives primarily focus on a single type of arthroplasty, particularly TKA. In contrast, our study encompasses both TKA and THA and includes a larger patient cohort. Consequently, our findings provide more comprehensive and substantial data to aid surgeons in clinical decision-making.^20-23^

 Since the previous meta-analysis, several new studies have been published on the impact of MBS on THA/TKA outcomes, highlighting that this topic remains a subject of ongoing debate.^24^ Therefore, in this systematic review and meta-analysis, we aimed to demonstrate the effects of prior MBS on complications following TKA/THA. Our primary hypothesis is that MBS will have a beneficial impact on the outcomes of TKA/THA.

## Materials and Methods

###  Search Strategy

 We followed the Preferred Reporting Items for Systematic Reviews and Meta-Analyses (PRISMA) guidelines for this systematic review and meta-analysis. Up to September 5, 2024, the following keywords were used to search PubMed, Embase, Scopus, and Web of Science: (“Bariatric” OR “Metabolic Surgery” OR “Stomach Stapling” OR “ Weight lose surgery “ OR “Obesity surgery” OR “Gastric Bypass” OR “Jejunoileal Bypass” OR “Roux-en-Y Gastric Bypass” OR “RYGB” OR “Gastric Sleeve” OR “Sleeve gastrectomy” OR “gastric banding” OR “biliopancreatic diversion” OR “duodenal switch” OR “duodenojejunal bypass” OR “jejunoileal bypass” OR ‘’Single anastomosis bypass’’ OR ‘’OAGB’’ OR ‘’mini bypass’’ OR ‘’one anastomosis Gastric bypass’’ OR ‘’Single loop Gastric bypass’’ OR ‘’Omega loop bypass’’ OR ‘’Omega loop gastric bypass’’) AND (“Knee Replacement Arthroplasties” OR “Knee Replacement Arthroplasty” OR “Total Knee Arthroplasty” OR “Total Knee Arthroplasties” OR “Total Knee Replacement” OR “Knee Arthroplasty” OR “TKR” OR “TKA” OR “knee replacement” OR “Hip Replacement Arthroplasty” OR “Hip Replacement Arthroplasties” OR “Hip Prosthesis Implantation” OR “Hip Prosthesis Implantations” OR “Total Hip Replacements” OR “Total Hip Replacement” OR “Total Hip Arthroplasty” OR “Total Hip Arthroplasties”). Additionally, the references of the included studies were used in the manual search.

###  Inclusion and Exclusion Criteria 

 We included all the retrospective and prospective cohort studies and randomized controlled trials (RCTs) that compare the outcomes of TKA/THA between two groups of patients. The case group contains patients with a history of different types of prior MBS, and the control group is patients with a BMI ≥ 30 kg/m^2^ and without a history of MBS at the time of TKA/THA. In addition, conference abstracts, case reports, protocols, editorials, reviews, animal studies, non-English language studies, studies that included total joint arthroplasties other than THA or TKA, and studies that focused only on non-surgical weight loss methods were excluded.

###  Study Selection

 After excluding duplicate articles, two independent authors screened the articles by titles and abstracts. Full-text reviews were conducted on pertinent articles. A third reviewer was consulted to settle any disagreements.

###  Quality Assessment

 For RCT studies, the Cochrane Risk of Bias tool 2 was employed. The quality of observational studies was evaluated using the Newcastle-Ottawa Scale (NOS). The included studies were evaluated by two separate reviewers, and any discrepancies were resolved by consulting a third reviewer. Accordingly, among our 13 included articles, we did not detect any high-risk studies. The overview and evaluation for every study are shown in Tables S1 and S2 (See Supplementary file 1).

###  Data Extraction

 Two separate authors extracted the following variables from the full texts of the included studies:

- Study characteristics: Title, first author, publication year, study design, country.
- Intervention details: Type of metabolic and bariatric surgery (MBS), joint arthroplasty procedure (TKA/THA).
- Demographics: Age, gender, sample size, follow-up duration, mean time between MBS and arthroplasty.
- Clinical metrics: Preoperative BMI, comorbidities.
- Postoperative outcomes: Revision, reoperation, deep venous thrombosis (DVT), pulmonary thromboembolism (PTE), wound complications, prosthetic joint infection (PJI), postoperative manipulation.

Discrepancies in data extraction were resolved through rechecking by a third author and further discussion. Studies with distinct patient cohorts or surgical protocols were treated as independent datasets in the quantitative analysis.

###  Outcome Measures

 The primary outcome of this study was to evaluate the impact of prior MBS on the outcomes of THA and TKA.

###  Statistical Analysis

 Mean ± standard deviations (SD) were used for continuous variables, whereas frequency was used for categorical variables. For the conversion of median and interquartile range or range to mean ± SD, we used the formulas presented by Hozo et al, Luo et al, and Wan et al ^25-27^ Stata/SE, version 17 (StataCorp LLC), was used for all quantitative analyses. I^2^ was computed to evaluate heterogeneity, and studies with an I^2^ greater than 50% were considered to be severely heterogeneous. For the severe and non-severe heterogeneous analyses, random and fixed effect model analyses were carried out, respectively. Stata provides the log odds ratio as the outcome of a pooled analysis of categorical variables. This formula is used in converting these log odds ratios to odds ratios: odds ratio = e^ (log odds ratio).* P* values less than 0.05 were considered statistically significant.

###  Leave-one-out Analysis

 We conducted leave-one-out sensitivity analyses across all meta-analyses to assess the robustness of pooled estimates.

###  Publication Bias 

 We performed trim-and-ﬁll and funnel plots to identify publication bias in our meta-analyses.

## Results

**Figure 1:**
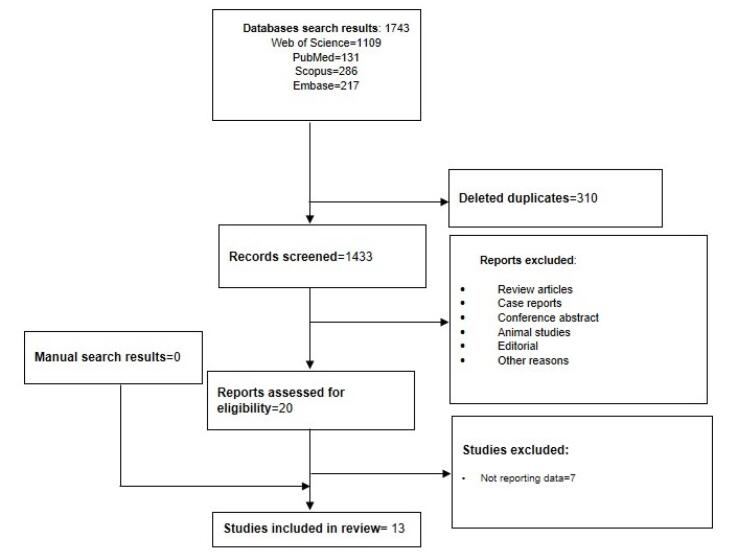
PRISMA (Preferred Reporting Items for Systematic reviews and Meta-Analyses) Flowchart Showing the Selection Process of Included Studies

 After a systematic search through PubMed, Scopus, Embase, and Web of Science databases, 1743 articles were found. After excluding 310 duplicate articles, the titles and abstracts of 1433 remaining articles were reviewed. The remaining papers were subjected to a full-text review after the title and abstract reviews. A total of 13 articles met the inclusion criteria for the final analysis. Among the selected studies, only one study was an RCT, and all others were retrospective studies. Table 1 provides a summary of the general characteristics of the included studies. Figure 1 shows the study’s PRISMA flowchart.

**Table 1:** Summary of Included Studies

**Author**	**Sample size**		**Age (years)**		**Gender** **(female)**		**Indications for TKA/THA**	**Type of MBS**	**Type of arthroplasty**	**Time between MBS to arthroplasty **	**Follow-up**	**MBS BMI** **VS ** **Non-MBS BMI before orthopedic surgery**
	**MBS**	**Non-MBS**	**MBS**	**Non-MBS**	**MBS**	**Non-MBS**						
A. Kulkarni^28^	90	53	57	56	-	-	-	Sleeve gastrectomy, Banding of stomach, Bypass of stomach by anastomosis of stomach to transposed jejunum	THA, TKA	At least 6 months	3-18 months	-
Erik P. Severson^29^	61	39	59 ± 8.4	55.5 ± 6.5	49	28	-	Gastric bypass, gastric banding	TKA	More than 2 years	22months- 14 years	38.5 ± 9.8 Vs 43.1 ± 6.3
Maria C.S. Inacio^30^	69	11032	59.9 ± 7.8	63.8 ± 8.7	52	7496	-	-	THA, TKA	More than 2 years	MBS: 320 ± 259 days Non-MBS: 1076 ± 717 days	34.6 ± 6.2 Vs 40.0 ± 4.4
J. R. Martin^31^	91	91	58.1 ± 8	57.4 ± 7	74	74	OA, post-traumatic	-	TKA	MBS: 46.5 months vs non-MBS: not applicable	MBS: 3.9 ± 1.8 years Non-MBS: 4.1 ± 2.2 years	37.2 ± 7 Vs 51.2 ± 9
C. D. Watts^32^	42	90	57.1 ± 12	56.5 ± 11	27	54	OA, post-traumatic	-	THA	5y (4 m - 12y)	3 years (2 to 9 years)	35.3 ± 7 Vs 50.2 ± 11
Philippe Hernigou^33^	79	200	71 ± 8	72 ± 9	48	117	Primary hip OA, Dysplasia OA, Osteonecrosis, Rheumatoid arthritis of the hip	-	THA	Within 2 years	MBS: 11 years Non-MBS: 18 years	27.6 ± 4.2 Vs 39.4 ± 5.0
Emanuel E. Nearing^19^	66	36	56.3 ± 6.5	55.0 ± 5.5	51	31	-	Roux-en-Y, sleeve gastrectomy	TKA, THA	MBS: 4.9 ± 3.2 Non-MBS: 4.3 ± 3.3	MBS: 3.2 ± 2.8 y Non-MBS: 9.2 ± 3.2 y	37.6 ± 7.4 Vs 43.7 ± 5.7
Jiabin Liu^18^	1894	1000	at first surgery: 58 ± 8.89	58 ± 7.41	1452	775	-	Roux-en-Y, sleeve gastrectomy	TKA, THA	Maximum 5 years	-	-
Sean P. Ryan^34^	205	205	62	62	168	168	-	Roux-en-Y, sleeve gastrectomy, laparoscopic bandings	TKA	11 y (range: 3 months - 44 years)	6 years (range, 2-20 years)	36.9 ± 7.2 vs 44.4 ± 4.1
Stephanie Purcell^35^	21	12	55.0 ± 7.2	43.2 ± 9.4	15	7	Knee pain	Laparoscopic sleeve gastrectomy	TKA	4.2 ± 1.9 years	from 1 month to 1 year	-
Michelle M. Dowsey^36^	41	41	58.7 ± 3.7	57.0 ± 5.7	32	34	-	Laparoscopic adjustable gastric banding	TKA	12 months	12 months	43.8 ± 4.8 vs 43.6 ± 6.3
Perna Ighani Arani^37^	465	119	55 ± 6.8	56 ± 5.7	349	91	OA	Gastric bypass or sleeve gastrectomy	TKA	13 months	MBS: 24 months Non-MBS: 39 months	31 ± 4.4 Vs 38 ± 4.6
David A. Momtaz^38^	451	451	56.33 ± 8.91	55.81 ± 13.73	313	320	-	Roux-en-Y, longitudinal gastrectomy	THA	1-5 years	Up to 72 months	35.61 ± 5.62 Vs 36.21 ± 6.32

TKA: total knee arthroplasty, THA: total hip arthroplasty, MBS: metabolic and bariatric surgery, HTN: hypertension, DM: diabetes mellitus, OA: osteoarthritis, COPD: chronic obstructive pulmonary disease.

 In total, MBS and non-MBS groups consisted of 3,575 and 13,369 patients, respectively. The mean follow-up period was in the range of one month to 18 years. Of the 13 studies included, four (31%) examined TKA and THA following MBS, six (46%) concentrated solely on TKA, and three studies (23%) only examined THA. Table 2 summarizes the results of the current study.

**Table 2:** Summary of Postoperative Complications Analysis between MBS and Non-MBS Groups

**Postoperative Complications**	**Number of Studies**	**Odds Ratio (95% CI)**	***P*** ** Value**	**I**^2^
Wound complication	9	0.68 (0.44, 1.05)	0.08	47.07%
DVT	8	0.50 (0.28, 0.86)	0.01	0.00%
PTE	5	0.96 (0.45, 2.01)	0.91	0.00%
Aseptic loosening	3	1.42 (0.41, 5.01)	0.58	22.65%
Blood transfusion	2	1.70 (0.11, 27.16)	0.71	64.71%
Dislocation	5	1.05 (0.62, 1.77)	0.87	0.00%
Peri-prosthetic fracture	3	2.94 (0.57, 15.07)	0.20	0.00%
PJI	5	0.55 (0.31, 0.97)	0.04	0.00%
Postoperative manipulation	4	1.48 (0.71, 3.07)	0.30	0.00%
30-day Readmission	3	0.33 (0.07, 1.63)	0.17	73.18%
Reoperation	6	1.63 (0.59, 4.53)	0.34	70.04%
Revision	8	1.15 (0.50, 2.69)	0.74	58.78%
LOS	6	Mean difference: -0.42 (-0.71, -0.13)	< 0.001	63.37%

DVT: deep venous thromboembolism, PTE: pulmonary thromboembolism, PJI: peri-prosthetic joint infection, LOS: length of stay.

###  Pre-arthroplasty BMI

**Figure 2:**
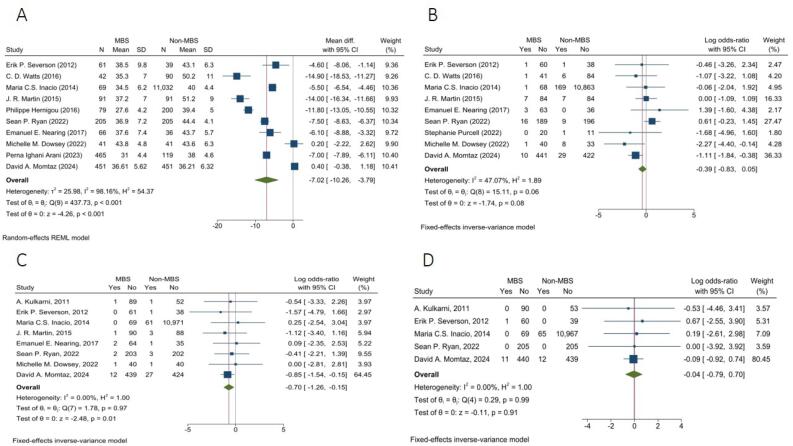
Forest Plots of BMI and Postoperative Complications between MBS and Non-MBS groups. (A) pre-operative BMI, (B) Wound complication, (C) DVT, and (D) PTE

 The random-effects model pooled analysis showed that the MBS group had significantly lower BMI with a mean difference of -7.02 kg/m^2^ in comparison to the non-MBS group (95% CI: -10.26, -3.79, I^2^ = 98.16%, *P* < 0.001) (Figure 2A).

###  Wound Complication

 The fixed-effects model pooled analysis of nine studies with 1047 and 11,997 patients in MBS and non-MBS groups demonstrated that the rate of wound complications was not significantly different between the two groups (OR = 0.68, 95% CI: 0.44, 1.05, I^2^: 47.07%, *P* = 0.08) (Figure 2B).

####  Deep Venous Thrombosis 

 The fixed-effects model pooled analysis of eight articles with 1074 and 11948 patients in MBS and non-MBS groups presented that the risk of DVT was significantly lower in the MBS group (OR = 0.50, 95% CI: 0.28, 0.86, I^2^: 0.00%, *P* = 0.01) (Figure 2C).

####  Pulmonary Thromboembolism 

 The fixed-effects model pooled analysis of five studies with 876 and 11,780 patients in the MBS and non-MBS groups showed that the rate of PTE was not significantly different between the two groups (OR = 0.96, 95% CI: 0.45, 2.01, I^2^: 0.00%, *P* = 0.91) (Figure 2D).

####  Aseptic Loosening 

**Figure 3:**
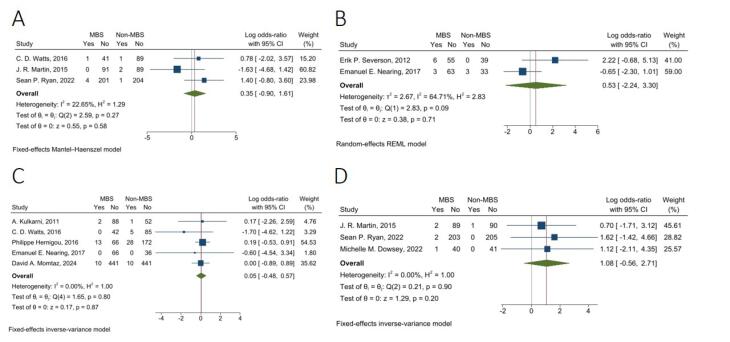
Forest Plots of Postoperative Complications Between MBS and Non-MBS Groups. (A) Aseptic loosening, (B) Blood transfusion, (C) Dislocation, and (D) Peri-prosthetic fracture

 The fixed-effects model pooled analysis of three studies with 338 and 386 patients in the MBS and non-MBS groups showed that the rate of aseptic loosening was not significantly different between the two groups (OR = 1.42, 95% CI: 0.41, 5.01, I^2^: 22.65%, *P* = 0.58) (Figure 3A).

####  Blood Transfusion

 The random-effects model pooled analysis of two studies with 127 and 75 in MBS and non-MBS groups demonstrated that the rate of blood transfusion was not significantly different between the two groups (OR = 1.70, 95% CI: 0.11, 27.16, I^2^: 64.71%, *P* = 0.71) (Figure 3B).

####  Dislocation 

 The fixed-effects model pooled analysis of five studies with 728 and 830 patients in MBS and non-MBS groups showed that the rate of joint dislocation was not significantly different between the two groups (OR = 1.05, 95% CI: 0.62, 1.77, I^2^: 0.00%, *P* = 0.87) (Figure 3C).

####  Peri-prosthetic fracture 

 The fixed-effects model pooled analysis of three studies with 337 and 337 patients in MBS and non-MBS groups showed that the rate of peri-prosthetic fracture was not significantly different between the two groups (OR = 2.94, 95% CI: 0.57, 15.07, I^2^: 0.00%, *P* = 0.20) (Figure 3D).

####  Prosthetic Joint Infection 

**Figure 4:**
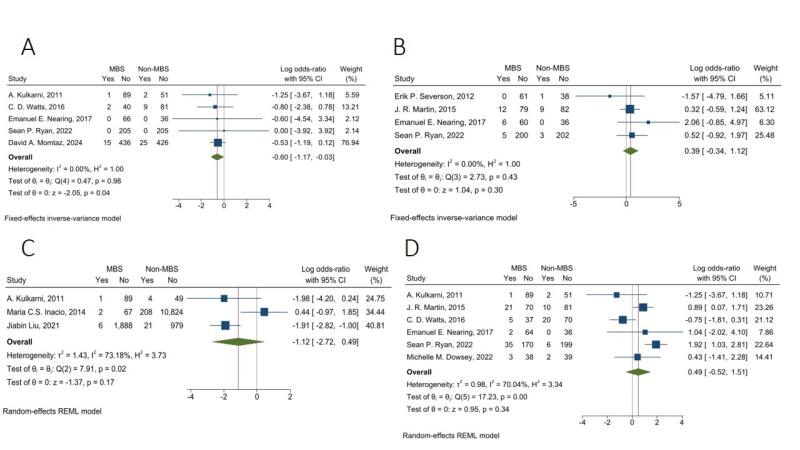
Forest Plots of Postoperative Complications Between MBS and Non-MBS Groups (A) Peri-prosthetic Joint Infection, (B) Postoperative Manipulation, (C) 30-Day Readmission, and (D) Reoperation

 The fixed-effects model pooled analysis of five studies with 853 and 835 patients in the MBS and non-MBS group presented that the rate of PJI was significantly lower in the MBS group compared to the non-MBS group (OR = 0.55, 95% CI: 0.31, 0.97, I^2^: 0.00%, *P* = 0.04) (Figure 4A).

####  Postoperative Manipulation

 The fixed-effects model pooled analysis of four studies with 423 and 371 patients in MBS and non-MBS groups demonstrated that the rate of postoperative manipulation was not significantly different between the two groups (OR = 1.48, 95% CI: 0.71, 3.07, I^2^: 0.00%, *P* = 0.30) (Figure 4B).

####  30-Day Readmission

 The random-effects model pooled analysis of three studies with 2,053 and 12,085 in MBS and non-MBS groups showed that the rate of 30-day readmission was not significantly different between the two groups (OR = 0.33, 95% CI: 0.07, 1.63, I^2^: 73.18%, *P* = 0.17) (Figure 4C).

####  Reoperation

 The random-effects model pooled analysis of six studies with 535 and 516 in MBS and non-MBS groups presented that the rate of reoperation was not significantly different between the two groups (OR = 1.63, 95% CI: 0.59, 4.53, I^2^: 70.04%, *P* = 0.34) (Figure 4D).

####  Revision

**Figure 5:**
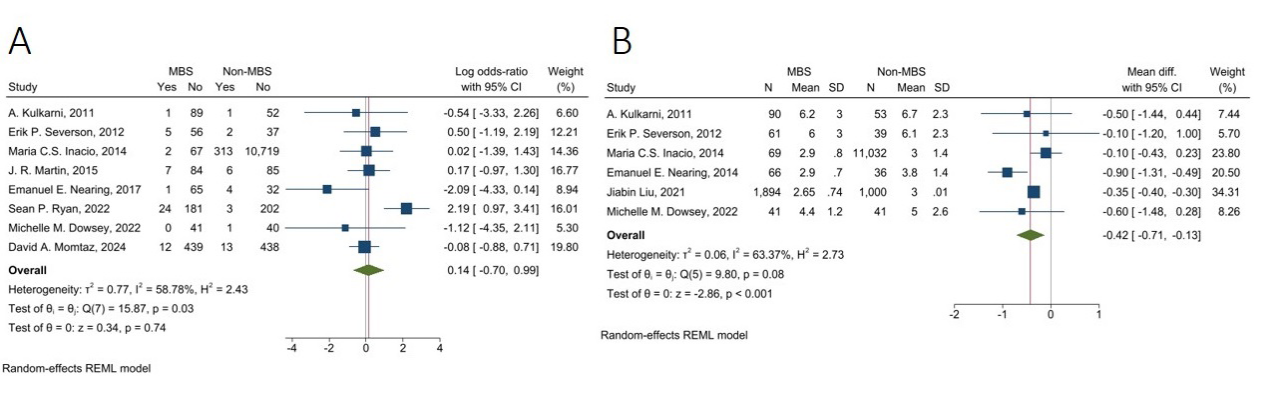
Forest Plots of Postoperative Complications Between MBS and Non-MBS Groups (A) Revision and (B) Length of Stay

 The random-effects model pooled analysis of eight studies, with 1074 and 11,948 in MBS and non-MBS groups showed that the rate of revision was not significantly different between the two groups (OR = 1.15, 95% CI: 0.50, 2.69, I^2^: 58.78%, *P* = 0.74) (Figure 5A).

####  Length of Hospital Stay 

 The random-effects model pooled analysis of six studies with 2,221 and 12,201 patients in the MBS and non-MBS groups showed that LOS in the hospital was significantly lower in the MBS group with a mean difference of -0.42 days (95% CI: -0.71, -0.13, I^2^: 63.37%, *P* < 0.001) (Figure 5B).

####  Quality Assessment

Tables S1 and S2 show the results of quality assessments of observational and RCT studies, respectively. The total risk of bias in the included RCT study was low. In addition, all of the included observational studies had a low or moderate risk of bias.

####  Leave-one-out Analysis

**Figure 6:**
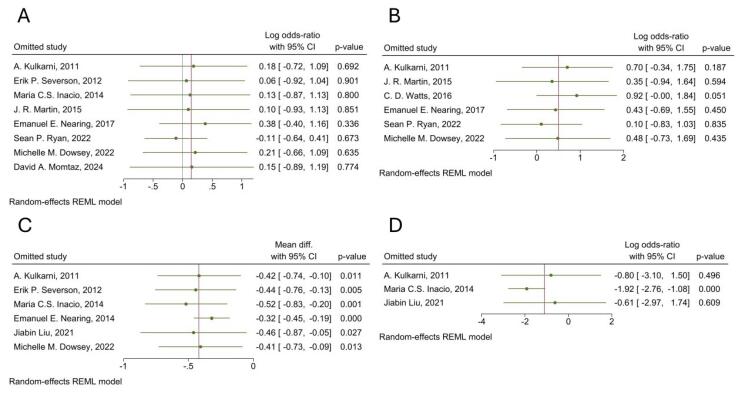
Leave-one-out Analysis of (A) Revision, (B) Reoperation, (C) LOS, and (D) 30-Day Readmission

 Leave-one-out sensitivity analyses revealed robust overall effect estimates across all variables except readmission. For outcomes demonstrating severe heterogeneity (revision, reoperation, and length of stay), the pooled estimates remained stable, with no single study exclusion substantially altering the magnitude or direction of effects (Figure 6A-C). In addition, when performing the leave-one-out sensitivity analysis for 30-day readmission, omission of Maria C.S. Inacio resulted in a significant pooled OR (OR = 0.15, 95% CI: 0.06, 0.34, *P* < 0.001), indicating that the overall findings are primarily influenced by the Maria C.S. Inacio, 2014 study (Figure 6D).

####  Publications Bias

 Both funnel plots and trim-and-fill analyses indicated no evidence of publication bias in our meta-analyses.

## Discussion

 The current study is a comprehensive analysis of how MBS affects the post-operative outcomes of TKA and THA compared to patients with obesity and without a history of MBS. This systematic review and meta-analysis included 13 studies with 22,191 patients. According to the findings, MBS was associated with a lower risk of PJI and DVT, and shorter LOS in patients who underwent TKA/THA.

 Patients with obesity are more likely to experience perioperative and intraoperative complications and adverse outcomes after TKA and THA. So, it is crucial to manage obesity before surgery.^3,6,13,15^ Due to the growing obesity epidemic, the prevalence of TJA is rising. Delaying arthroplasty in patients with severe obesity may be beneficial, as weight loss treatments like MBS can play a supportive role. As a result, bariatric and orthopedic surgeons will remain essential in the comprehensive care of patients with obesity. Nevertheless, the risks of subsequent TKA/THA with and without prior MBS are still a topic of question.^6,7,17,22,24^

 In our meta-analysis of THA/TKA outcomes, results show that the rate of complications after THA/TKA, including PJI and DVT, was 45% and 50% lower in patients with prior MBS compared to non-MBS, respectively. In addition, LOS was significantly shorter with a mean difference of 0.42 days in patients with prior MBS compared to non-MBS. However, other complications, including aseptic loosening, blood transfusion, wound complications, PTE, dislocation, peri-prosthetic fracture, postoperative manipulation, 30-day readmission, reoperation, and revision, were not significantly different between the two groups.

 Obesity significantly raises the risk of DVT, and there is a dose-response association between rising DVT risk and rising BMI.^39,40^ Pro-coagulant factors and fibrinolysis impairment significantly increase with BMI. Possible pathophysiological mechanisms like elevated levels of several pro-coagulant factors, venous stasis, chronic inflammation, and increasing activated protein C resistance with increasing BMI, either separately or in combination, may be important risk variables for DVT in patients with obesity.^39-41^ Therefore, managing obesity seems to mitigate the risk of DVT. Previous studies showed that MBS can significantly decrease the risk of DVT in patients with obesity.^42,43^ In our meta-analysis, MBS was associated with 50% reduced risk of DVT in patients who underwent TKA/THA compared to the non-MBS group. However, there was no significant difference in PTE rates between the two groups. In contrast to our results, a previous meta-analysis by Li et al found no significant difference in short-term DVT following TJA between MBS and non-MBS groups.^22^ Furthermore, Smith et al in 2016 did not find significant differences between MBS and non-MBS groups in terms of DVT (RR 0.57; 95% CI 0.13 to 2.44) or PTE (RR 0.51; 95% CI 0.03 to 8.26) in patients who underwent TKA/THA.^21^ However, their findings are constrained by the limited and fewer number of studies. Additionally, our study includes a larger number of recent studies with lower heterogeneity, providing a more up-to-date and comprehensive analysis.

 Obesity increases the incidence of acute PJI following primary THA, independently of other comorbid conditions.^44,45^ It was noted that all obesity classes can raise the risk of PJI considerably.^46^ Even after receiving treatment for PJI, individuals with severe obesity have a higher rate of reinfection than those without obesity.^47^ Surgeons must take into consideration that healthy patients with a BMI over 40 have a 3 to 9-fold higher relative risk of PJI in long-term follow-up.^45^ Our results showed that the risk of PJI was 45% lower in the MBS group compared to the non-MBS group in patients who underwent TKA/THA. In line with our results, Mauro et al showed that MBS was associated with lower risk of PJI in patients who underwent TKA.^48^ The pathophysiology of obesity and increased risk of infection can be explained by the fact that there is a connection between immune-competent cells and adipocytokines that are released from the adipose tissue, due to excess adiposity, which is caused by its malfunction and reduced immune function in the presence of obesity. Patients who are obese have a higher prevalence and severity of infectious disease than lean patients.^49-51^ Therefore, it is advisable to manage obesity in patients undergoing TJA to minimize the risk of PJI.

 The healthcare system could be heavily burdened by longer LOS. Obesity prevention and treatment will probably result in fewer hospitalizations and lower healthcare charges related to the obesity epidemic.^52,53^ An association between obesity and longer LOS has been shown in different surgical patients.^54,55^ It was demonstrated that obesity is associated with longer LOS and cost of hospitalization in patients who underwent THA.^56^ Our results showed that LOS was significantly lower in the MBS group, with a mean difference of -0.42 days in patients who underwent TKA/THA.

 Even though revision THA can effectively treat a failed initial hip arthroplasty, patients with obesity who have this treatment will experience greater revision and complication rates, especially dislocation, compared to non-obese patients.^57^ Also, patients with severe obesity were four times more likely to need primary revision after TKA because of dislocation compared to the non-obese population. Severe obesity was also discovered to be an independent predictor of implant loosening. Additionally, patients with morbid obesity had a higher risk of malposition and stiffness.^58^ However, our results did not show a significant difference in terms of revision, dislocation, and aseptic joint loosening between the MBS and non-MBS groups.

 This meta-analysis has several limitations. First, some of our non-significant variables like blood transfusion, 30-d readmission, reoperation, and revision had high heterogeneity. However, to strengthen our findings, we performed a leave-one-out sensitivity analysis. Also, none of the studies discussed nutrient insufficiency in patients with a history of MBSs, although this can affect some postoperative outcomes and possibly some of the postoperative complications that occur due to this nutrient insufficiency. Most articles did not report the exact number of patients with their specific type of MBS, so due to inadequate data, we were unable to do a subgroup analysis based on the type of prior MBS. In addition, the majority of the studies were retrospective, and we had only one RCT; this can increase the risk of selection and recall bias. Additionally, details regarding surgical techniques, perioperative management, and the criteria for revision surgery were limited. Future RCT studies are recommended to compare the impact of different modalities of obesity management (medical vs. surgical) in patients who undergo TKA/THA.

## Conclusion

 Our study showed that MBS was significantly associated with reduced risk of DVT and PJI, as well as shorter LOS in patients who underwent TKA/THA. However, the rate of aseptic loosening, blood transfusion, wound complications, PTE, dislocation, peri-prosthetic fracture, postoperative manipulation, 30-day readmission, reoperation, and revision did not differ significantly between the MBS and non-MBS groups. MBS can be considered as a viable option not only for managing obesity but also for reducing postoperative complications in patients with severe obesity who are candidates for TKA/THA.

## Supplementary Files

Supplementary file 1 contains Tables S1 and S2.
