# Supplementary material for: Impact of Prior Metabolic and Bariatric Surgery on Outcomes of Total Knee and Total Hip Arthroplasty: A Systematic Review and Meta-analysis
Source: Arch Iran Med. 2025 Aug 1;28(8):461–71. doi: 10.34172/aim.34291 (PMC12569990; doi:10.34172/aim.34291)
Supplement: Supplementary file 1 — contains Tables S1 and S2. [file aim-28-461-s001.pdf]

Arch Iran Med. August 2025;28(8):461-S1

10.34172/aim.34291

http://journalaim.com

Supplementary Files

Table S1. Quality assessment of cohort studies using the Newcastle-Ottawa Quality Assessment Scales.

**Items**		**A. Kulkarni 2011**	**Erik P. Severson 2012**	**Maria C.S. Inacio 2014**	**J. R. Martin 2015**	**C. D. Watts 2016**	**Philippe Hernigou 2016**	**Emanuel E. Nearing 2017**	**Jiabin Liu 2021**	**Sean P. Ryan 2022**	**Stephanie Purcell 2022**	**Perna Ighani Arani 2023**	**David A. Momtaz 2024**
**Selection**	**Representativeness of the exposed cohort**	A (star)	A (star)	A (star)	A (star)	A (star)	B (star)	A(star)	A(star)	A(star)	A(star)	A(star)	A(star)
	**Selection of the non-exposed cohort**	A (star)	A (star)	A (star)	A (star)	A (star)	No star	A (star)	A(star)	A(star)	A(star)	A(star)	A(star)
	**Ascertainment of exposure**	A (star)	A (star)	A (star)	A (star)	A (star)	A (star)	A(star)	A(star)	A(star)	A(star)	A(star)	A(star)
	**Demonstration that outcome of interest was not present at start of study**	A (star)	A (star)	A (star)	A (star)	A (star)	A (star)	A(star)	A(star)	A(star)	A(star)	A(star)	A(star)
**Comparability**	**Comparability of cohorts on the basis of the design or analysis**	A (star)	No star	No star	A (star)	B (star)	No star	No star	B (star)	B(star)	No star	No Star	B (star)
**Exposure**	**Assessment of outcome**	B (star)	B (star)	B (star)	B (star)	B (star)	B (star)	B (star)	B (star)	B (star)	B (star)	B (star)	B (star)
	**Was follow-up long enough for outcomes to occur?**	A (star)	A (star)	A (star)	A (star)	A (star)	A (star)	A(star)	No Star	A(star)	No star	A(star)	A(star)
	**Adequacy of follow up of cohorts**	D	A (star)	B (star)	D	D	B (star)	D	No Star	B(star)	B(star)	B(star)	B(star)
**Overall risk of bias**		Moderate	Moderate	Moderate	Moderate	Moderate	Moderate	Moderate	Moderate	Low	Moderate	Moderate	Low

Table S2. Quality assessment of RCT study using the Cochrane risk-of-bias tool 2.

Items	Dowsey, 2022
**Risk of bias arising from the randomization process**	Low risk
**Effect of assignment to intervention**	Low risk
**Risk of bias due to missing outcome data**	Low risk
**Risk of bias in measurement of the outcome**	Low risk
**Risk of bias in selection of the reported result**	Low risk
**Overall**	Low risk
